# Marginal socio-economic effects of an employer’s efforts to improve the work environment

**DOI:** 10.1186/s40557-018-0212-5

**Published:** 2018-02-01

**Authors:** Mahmoud Rezagholi

**Affiliations:** Department of Business and Economic Studies, Division of Economics, University of Gävle, -801 76 Gävle, SE Sweden

**Keywords:** Workplace health promotion, Employer’s multifactorial efforts, Overall labour effectiveness, Informal work impairments, Labour productivity loss, Illness-related lost working hours

## Abstract

**Background:**

Workplace health promotion (WHP) strongly requires the employer’s efforts to improve the psychosocial, ergonomic, and physical environments of the workplace. There are many studies discussing the socio-economic advantage of WHP intervention programmes and thus the internal and external factors motivating employers to implement and integrate such programmes. However, the socio-economic impacts of the employer’s multifactorial efforts to improve the work environment need to be adequately assessed.

**Methods:**

Data were collected from Swedish company Sandvik Materials Technology (SMT) through a work environment survey in April 2014. Different regression equations were analysed to assess marginal effects of the employer’s efforts on overall labour effectiveness (OLE), informal work impairments (IWI), lost working hours (LWH), and labour productivity loss (LPL) in terms of money.

**Results:**

The employer’s multifactorial efforts resulted in increasing OLE, decreasing IWI and illness-related LWH, and cost savings in terms of decreasing LPL.

**Conclusion:**

Environmental factors at the workplace are the important determinant factor for OLE, and the latter is where socio-economic impacts of the employer’s efforts primarily manifest.

## Background

Satisfied and productive employees are necessary to create a successful company, while dissatisfied and unproductive workers jeopardize the company’s image, sustainability, competitiveness, and survival [1]. The well-being and productivity of the workforce is conditional on a well-functioning work environment with minimum risk factors for illness [1–6]. Many disorders in a company are related not only to the physical environment at the workplace [2–7], but also to the work conditions along a series of psychosocial and ergonomic factors that characterize the work culture in the company [1–6, 8–13]. Efforts to create a pleasant and productive work environment can be made by employers, employees, and even society’s health policy makers. The term *workplace health promotion* (WHP) is thus defined by the European Network for Workplace Health Promotion (ENWHP) in the Luxemburg Declaration 2007 as the ‘combined efforts’ of employers, employees, and society to improve the mental and physical health and well-being of people at work (http://www.enwhp.org/fileadmin/rs-dokumente/dateien/Luxembourg_Declaration.pdf). However, management plays a crucial role in these combined efforts when it can lend its full support for effective WHP programmes to be processed, integrated into company health policy, and allocated sufficient financial and physical resources [14]. Everything from optimization of the physical environment at the workplace for different work functions up to the improvement of ergonomic and psychosocial working conditions can increase workers’ health and well-being and thereby the company’s sustainability and future benefits. WHP intervention programmes have been shown to result in reduced stress and physical strain [14], less sick leave, improved psychosocial well-being and work satisfaction [15], better social support from supervisors and colleagues [16], and also reduced cost for work-related disorders [1, 17]. Thus, there are many factors motivating employers to implement and integrate WHP programmes. These include factors relating to the economic costs saved by reducing sickness absenteeism and presenteeism, and thus production loss; but also, those relating to the economic benefits of increasing workers’ potential productivity [1]. There are also external motivating factors such as improved corporate image and customer loyalty, and increased governmental and insurance support [14].

The social costs of work-related disorders, the socio-economic impacts of work environmental risk factors, and even the economic value of the information to be produced during proposed work environmental studies have recently been assessed using appropriate economic theories and methodologies [1, 2, 17]. However, the employer’s efforts and their socio-economic consequences have not yet been assessed in the same manner. Employers’ multifactorial efforts consist of ‘rewards’ as compensation for workers’ efforts. These rewards are mostly represented by money, self-esteem, career opportunity, and job security [18]; but also by improved organizational support [19] and optimized physical and ergonomic environmental factors [7, 10]. The literature referenced above advise employers to create and promote a healthy, well-functioning and productive environment for a safe workplace. The literature argue for:

1. Optimizing physical factors such as lighting and noise [7, 20];
2. Improving ergonomic working conditions [8–10];
3. Balancing of job demands and job control [21, 22];
4. Rotation of tasks to break the boring monotony [23–25];
5. Career opportunity as an important psychosocial reward [18, 26];
6. Appreciating and rewarding employees’ challenging work [18, 21, 27, 28];
7. Perceived organizational and supervisor support [19];
8. Adequate earnings and job security [29].

The objective of this article is to assess the socio-economic consequences of an employer’s efforts to improve the above-mentioned environmental factors at the workplace. This includes the addressing of several issues: the marginal effects of the multifactorial efforts on labour efficiency, work impairments, and lost working hours; the marginal cost of labour inefficiency; and the economic benefits of the employer’s efforts.

## Methods

### Data collection and basic concepts

All data used in this study were collected from a work environmental survey in the main plant of the Swedish company Sandvik Materials Technology (SMT) during May 2015. The collected dataset included data about work capacity, work performance, and work quality in addition to data on lost working hours due to sickness absenteeism and presenteeism. The employer’s efforts to improve the work environment are described in Table 1 in terms of psychosocial, ergonomic, and physical factors.

**Table 1:** Specification of the employer’s efforts to optimize factors in the psychosocial, ergonomic, and physical dimensions of the work environment

Psychosocial factors to be optimized/adjusted	Ergonomic factors to be optimized/adjusted	Physical factors to be optimized/adjusted
Job demand	Body postures while performing tasks	Air quality
Expectations of employees	Job monotony and job rotation	Noise
Organizational changes and stability	Handling heavy objects	Temperature
Status and carrier opportunities		Vibration
Conflict resolution		Lighting
Job security		
Salary and rewards		
Agreement		
Gratitude		
Principle of equal treatment and equal rights		
Job control		
Resolution of violent behaviours (insults, bullying, and harassment)		

A total of 49 workers participated anonymously in the study. Most of them were involved with manufacturing and were sufficient qualified for and experienced in their tasks. Some of the collected data have been used previously in other studies [1, 2, 17]. The questionnaire, which was distributed to the workers by the SMT safety unit during April 2015, was based on the Harvard questionnaire (http://www.hcp.med.harvard.edu/hpq/ftpdir/absenteeism%20presenteeism%20scoring%20050107.pdf), (http://www.hcp.med.harvard.edu/hpq/ftpdir/survey_clinical_7day.pdf), knowledge of Swedish workplaces and the Swedish labour market, and findings from the literature. As the employer’s multifactorial *efforts* to improve the work environment (EE) was difficult to assess directly, workers’ *satisfaction* with the employer’s efforts was used as an *indicator* of the level of these efforts in SMT. In the survey, the workers were asked to evaluate their physical, ergonomic, and psychological work environment. The workers were also asked to answer how they perceived their employer’s multifactorial efforts to improve the work environment. The answers would be in percent. To avoid missing and confusing answers, the participants answered the questions while the supervisor was in attendance. Then, the employer’s efforts were identical to the workers’ satisfaction with these efforts. For instance, if the workers were 70% satisfied with their employer’s efforts to improve the ergonomic work conditions, the efforts were assessed to be 70% of their potential level.

The other basic concepts were:

1. *Overall labour effectiveness* (OLE) defined as a product of the workforce *availability*, *work performance*, and *work quality*, measured in percentage units according to the following formula:

$$ \mathrm{OLE}=\left(\frac{\mathrm{actual}\  \mathrm{working}\  \mathrm{hours}}{\mathrm{scheduled}\  \mathrm{working}\  \mathrm{hours}}.\frac{\mathrm{actual}\  \mathrm{output}}{\mathrm{standard}\  \mathrm{output}}\cdot \frac{\mathrm{accepted}\  \mathrm{output}}{\mathrm{total}\  \mathrm{output}}\right)\cdot 100 $$

To assess the workforce availability, the workers were asked about their actual working hours compared to the scheduled working hours; to assess the work performance (i.e. the actual output divided to the standard output), the workers were asked about the achieved *quantitative requirements* of work in percent; and to assess the work quality (i.e. the accepted output divide to the total output), the workers were asked about the achieved *qualitative requirements* of work in percent. Both the company’s standard and the workers’ own judgements were considered in the assessments of the performance and the quality of work.

2) Indirect costs of sickness absenteeism and presenteeism in terms of *lost working hours* and *decreased labour productivity*. The costs were assessed in the basis of the registered illness-related lost working hours, the workers’ judgements about their working hours they worked at reduced work ability due to the environmental failures at the workplace, the national median wages, the rate of work interdependence, and the rate of unemployment and monopsony power [1].
3) *Informal work impairments*, which have previously been shown to be the largest source of the cost of work-related disorders in SMT [1, 17]. The company had registered data on the workers worked with reduced work ability in terms of part-time working, but not about the hours workers had been at work even though they, according to their health state, should had stayed at home. The non-registered work impairments were considered as informal. The informal work impairments also contained the time workers worked at reduced work *interest* and *motivation* due to the failures in the entire work environment.

### Analytical tools

Five regression equations were used to analyse the socio-economic consequences of labour ineffectiveness and the employer’s efforts to improve the work environment in SMT from May 2014 to April 2015. The socio-economic consequences were assessed in terms of important econometric issues. First, two regression models were used to assess the economic cost of labour ineffectiveness, which is measured as 1 – OLE, and the economic benefit of the employer’s efforts to improve the work environment. Then, three regression models were employed to assess the marginal effects of the employer’s efforts on labour effectiveness, informal impairments, and illness-related lost working hours.

The economic cost of labour ineffectiveness (LIE) in terms of labour productivity loss (LPL) was assessed by:

1 $$ \mathrm{LPL}={\upalpha}_1+{\upbeta}_2\cdot \mathrm{LIE}, $$

where LPL is expressed in terms of money, LIE is measured in percent, the intercept α_1_ is interpreted as the level of LPL independent of LIE, and β_1_ is the marginal cost of LIE (i.e. the cost of an additional percentage unit of LIE).

The economic benefit of the employer’s efforts to improve the work environment (EE) in terms of saving/decreasing labour productivity loss (LPL) was assessed by:

2 $$ \mathrm{LPL}={\upalpha}_2+{\upbeta}_2\cdot \mathrm{EE}, $$

where the intercept α_2_ is interpreted as the level of LPL independent of EE, and β_2_ is the marginal benefits of EE in terms of reducing LPL (i.e. the economic benefit of increasing EE by one precentage unit).

The impact of the employer’s efforts to improve the work environment (EE) on overall labour effectiveness (OLE) was assessed by:

3 $$ \mathrm{OLE}={\upalpha}_3+{\upbeta}_3\cdot \mathrm{EE}, $$

where both OLE and EE are measured in percentage units, the intercept α_3_ is interpreted as the level of OLE independent of EE, and β_3_ is the marginal effect of EE on OLE (also known as the OLE elasticity of EE).

The impact of the employer’s efforts to improve the work environment (EE) on informal work impairments (IWI); that is, reduced work capacity among workers which is not reflected in formal terms such as part-time working, was assessed by:

4 $$ \mathrm{IWI}={\upalpha}_4+{\upbeta}_4\cdot \mathrm{EE}, $$

where both IWI and EE are measured in percentage units, the intercept α_4_ is interpreted as the level of IWI independent of EE, and β_4_ is the marginal effect of EE on IWI (and known as the IWI elasticity of EE).

The impact of the employer’s efforts to improve the work environment (EE) on the working hours lost by ill workers (LWH) was assessed by:

5 $$ \mathrm{LWH}={\upalpha}_5+{\upbeta}_5\cdot \mathrm{EE}, $$

where the intercept α_5_ is interpreted as the amount of LWH independent on the level of EE, and β_5_ is the marginal effect of EE on LWH (i.e. the responsiveness of LWH to changes in EE by one percentage unit).

Ordinary least squares (OLS) regression analysis was used to assess the important marginal values.

## Results

The empirical results of applying the above-described regression equations at SMT during 2014–2015 are presented in the following subsections. All costs are in Swedish Crowns (SEK) and rounded to integers. Euro and US Dollar to SEK exchange rates as of 15 October 2015 were 9.30 and 8.12, respectively.

### The economic cost of labour ineffectiveness

The marginal cost of labour ineffectiveness in terms of labour productivity loss was estimated to be 6647 SEK on average for each SMT worker during 2014–2015. This means that each additional percentage unit of ineffectiveness embodied in a worker (due to individual characteristics, insufficient skills, and non-optimized environmental factors at the workplace) cost SMT 6647 SEK on average during that period. The autonomous cost of 50,665 SEK is the estimated cost not caused by workforce ineffectiveness. As an overall evaluation of the regression model, 58% of the variation in labour productivity loss among SMT workers during the study period can be explained by fluctuations in the labour ineffectiveness.

### The economic benefit of work environmental efforts

The marginal benefit (economic advantage) of the employer’s efforts in terms of decreasing illness-related labour productivity loss was estimated as 6241 SEK, meaning that SMT saved an average of 6241 SEK of productivity loss per worker during 2014–2015 for each additional percentage unit of its efforts to improve the work environment. The autonomous productivity loss of 637,895 SEK is the estimated productivity loss that cannot be saved by increasing the efforts. According to the regression model, 75% of the variation in labour productivity loss among the workers can be explained by fluctuations in the work environmental efforts.

### The effects of the employer’s efforts on overall labour effectiveness

The marginal effect of the employer’s efforts on overall labour effectiveness was 1.09, meaning that each additional percentage unit of the employer’s efforts increased the labour effectiveness by more than one percent. The employer’s efforts in SMT are thus characterized by a (weak) increasing return to labour effectiveness, which confirms the economic advantage in expansion of the efforts. The autonomous labour effectiveness not explained by the efforts was not statistically significant. However, 72% of the variation in labour effectiveness among the workers of SMT during 2014–2015 can be explained by fluctuations in the work environmental efforts.

### The effects of the employer’s efforts on workers’ informal impairments

The marginal effect of the employer’s work environmental efforts on workers’ informal impairments in SMT during 2014–2015 was − 0.69, meaning that the informal impairments decreased by 0.69% for each additional percentage unit of the efforts. The autonomous impairments, which cannot be explained by these efforts, were 67.63% of the total. The regression analysis showed that 73% of the variation in labour informal work impairments at SMT during 2014–2015 can be explained by fluctuations in the work environmental efforts.

### The effects of employer’s efforts on lost working hours

The marginal effect of the employer’s efforts to improve the work environment on the workers’ illness-related lost working hours was − 14.22, meaning that during 2014–2015 SMT could decrease workers’ illness-related LWH by 14.22 man-hours for each additional percentage unit of their efforts to improve the work environment. The estimated number of 1460 h reflects external factors affecting the amount of illness-related LWH, since 35% of the variation in workers’ LWH cannot be explained by fluctuations in EE.

### Summary of the results

The empirical results are summarized in Table 2, for an entire economic evaluation of the employer’s efforts to improve the work environment.

**Table 2:** The socio-economic consequences of labour ineffectiveness and the employer’s efforts to improve the work environment in SMT during 2014–2015

	Autonomous effect (*P*-value)	Marginal effect (*P*-value)
The effects of employer’s efforts on overall labour effectiveness	−10.69% (1.00)	1.09% (0.00)
The effects of employer’s efforts on informal impairments	67.63% (0.00)	−0.69% (0.00)
The effects of employer’s efforts on illness-related lost working hours	1460 h (0.00)	−14.22 h (0.00)
The effects of employer’s efforts on labour productivity loss	637,895 SEK (0.00)	−6241 SEK (0.00)
The effects of labour ineffectiveness on labour productivity loss	50,665 SEK (0.09)	6647 SEK (0.00)

## Discussions

### The *challenge* of improving work effectiveness

The challenge in the management of manufacturing organizations, in many countries, is to improve OLE as a measure for the cumulative effect of availability, work performance, and work quality [30, 31]. The managers are usually focused on factors as machinery, attendance and quality control, and professional knowledge and task training. However, previous research has definitely shown that there are other opportunities to optimize OLE; these consist of efforts aimed at optimizing environmental factors that affect the mental, social, and physical well-being of the workforce: workers’ attitudes and behaviours have a significant effect on their work performance and the production process [32, 33]; and lack of motivation affects the labour effectiveness not only directly, but also indirectly through the workers’ knowledge and skills [30]. Production rate and labour effectiveness are also affected by health and safety of the labour force, ergonomic work conditions [9, 10], ‘extrinsic rewards’ [29], and environmental factors such as noise, lighting, humidity, and temperature [7, 20]. The empirical results of this study have strengthen the early findings and assessed the effects of environmental factors at the workplace on OLE by using regression technique and economic theories; factors concerning the psychosocial, ergonomic, and physical dimensions of the work environment. Occupational health and job satisfaction are the most important sources of labour effectiveness, and can also strengthen the effectiveness of the traditional measures to increase availability, work performance, and product quality (i.e. efforts to improve knowledge, skills, and control systems for workers’ attendance and work quality). Workers’ health, well-being, and satisfaction can be improved by an investment in changing the psychosocial, ergonomic, and physical environments at the workplace; the cost of such an investment will be much less than its opportunity cost in terms of the costs of work-related disorders [1]. Thus, the employer’s efforts to improve the work environment and occupational health cannot be less effective and profitable than the abovementioned measures applied in the manufacturing industries, when the huge socio-economic costs of the work environmental risk factors for illness and impairments consist substantially of labour productivity losses [1, 3–6].

### Economic consequences of labour ineffectiveness and employer’s efforts

Both the marginal economic cost of labour ineffectiveness (6647 SEK) and the marginal economic benefit of employer’s efforts to improve the work environment (6241 SEK) were assessed in terms of the labour productivity loss (LPL). LPL was estimated on the basis of workers’ sickness absenteeism and presenteeism. A comparison analysis of the estimated marginal values showed that the labour ineffectiveness was substantially caused by the environmental risk factors at the SMT workplace, and that the effects of individual characteristics and insufficient qualification/experience were negligible. A major part of the company’s illness-related economic costs during the study period was due to the labour ineffectiveness caused by insufficiencies in the work environment. The association of OLE and EE in the regression model (3) explains why the difference between the marginal economic cost of labour ineffectiveness and the marginal benefit of the work environmental efforts was not considerable.

### The effects of *external factors* and necessary *assumptions*

In development of the models introduced here it was assumed that the business relationships and technical performance remained unchanged and were not subject to any unexpected anomalies and defects affecting labour productivity and effectiveness. This means assuming that 1) machinery was well maintained and in good condition, 2) raw and intermediate materials were supplied and delivered in good time and of the desired quality, 3) the relevant markets were in equilibrium, meaning that the related supply and demand curves remained unchanged, and 4) the level of workers’ qualification for and experience in their tasks remained constant during 2014–2015.

### *Obscurities* in the estimated autonomous effects

As usual in the econometrics literature, it must be emphasized that the estimated autonomous effects (effects not determined in the models) in these econometric analyses should not be used in decision-making regarding the work environment. In principle, they are either statistically non-significant or unexplainable and unrealistic. The estimated constant cost not explained by labour ineffectiveness (50,665 SEK) could be caused by sickness absenteeism, but the value has a weak significance level; the autonomous productivity loss not saved by work environmental efforts (637,895 SEK) is significant and caused by external factors, but is unrealistically high; the autonomous labour effectiveness with negative sign (− 10.69%) is non-significant, unexplainable, and unrealistic; the worker’s autonomous informal impairments (67.63%) show more realistically the combined effects of individual characteristics, societal factors, and the environmental factors at the workplace omitted in this study; and finally, the autonomous lost working hours (1460 h) are unexplained since they are much higher than the average yearly working hours lost by a worker (465 h).

### Differential marginal effects of the employer’s efforts – *recommendations* and *guidelines* for future research

The employer’s effort to improve the work environment was found to have an increasing but weak return to labour effectiveness, and a decreasing return to the workers’ informal impairments. Although the differential effects need to be addressed in future research, the results of this study and other studies based on the same dataset [1, 2] may allow the formation of some hypotheses around this; hypotheses to be tested in future research. The first is about the effect of *time*. The work environmental efforts have short-term effects on labour effectiveness, while their effects on work impairments are seen in the medium and long run. Thus, a long-run study of the efforts may smooth out the differential effects. Further, the employer’s efforts to improve the work environment have an impact not only on the quantity of work but also on the quality of work; this latter is reflected in the overall labour effectiveness but not in the work impairments. Moreover, a major part of the labour effectiveness can be explained by work environmental factors, compared with the illness-related informal work impairments.

The marginal effect of the employer’s efforts to improve the work environment on working hours lost by ill workers in SMT during 2014–2015 (14.22 h) indicates a strong potential ability of the efforts to reduce illness-related labour productivity loss in the company during this period. Each SMT worker lost 465 working hours on average during the study period due to sickness absenteeism and informal work impairments [1, 2]. This potential ability is verified by the high marginal economic benefit of the efforts (near the marginal economic cost of labour ineffectiveness) and their increasing return to overall labour effectiveness.

## Conclusions

This employer’s multifactorial efforts to improve the work environment have large and statistically significant socio-economic impacts in terms of increasing overall labour effectiveness and decreasing illness-related lost working hours, informal work impairments, and economic costs in terms of labour productivity loss. The impacts primarily manifest in the overall labour efficiency, and thus in informal work impairments and illness-related productivity loss.

## Abbreviations

EE: Employer’s Efforts (to improve the work environment)
ENWHP: European Network for Workplace Health Promotion
IWI: Informal Work Impairments
LIE: Labour Ineffectiveness
LPL: Labour Productivity Loss
LWH: Lost Working Hours
OLE: Overall Labour Effectiveness
SEK: Swedish Crowns
SMT: Sandvik Materials Technology
WHP: Workplace Health Promotion
